# Supplementary material for: Mapping of sex work hotspots to guide targeted HIV prevention: Evidence from eight Ukrainian cities
Source: PLoS One. 2026 Feb 26;21(2):e0343803. doi: 10.1371/journal.pone.0343803 (PMC12944754; doi:10.1371/journal.pone.0343803)
Supplement: S1 File — (PDF) [file pone.0343803.s001.pdf]

S1 File. Data Collection Tool – Hotspot Passport

Passport No.		City	

№	Question	Level 1<br>**Mapping**<br>*(information)*	Level 2<br>**Validation**<br>*(If the mapping data is confirmed, put +, if not confirmed, enter only the verified information)*
1	Form filling date		
2	Interviewer name		
3	Hotspot number from the database	Determined at L2	
4	Key informant number		
5	Key informant type **		
6	Administrative area		
**QUESTIONS ABOUT THE SW HOTSPOT**			
7	City district		
8	Hotspot address/web page		
9	Hotspot name (if available), incl. unofficial name		
10	Location of the sex workers (e.g., near hotel entrance)		
11	Hotspot type*		
12	Is the hotspot active in spring?	1 – yes, 2 – no	1 – yes, 2 – no
13	Is the hotspot active in summer?	1 – yes, 2 – no	1 – yes, 2 – no
14	Is the hotspot active in autumn?	1 – yes, 2 – no	1 – yes, 2 – no
15	Is the hotspot active in winter?	1 – yes, 2 – no	1 – yes, 2 – no
16	Hotspot operating schedule – indicate the working hours for each day		
	Monday	1 – yes, __:__ - __:__, 2 - no	1 – yes, __:__ - __:__, 2 – no
	Tuesday	1 – yes, __:__ - __:__, 2 - no	1 – yes, __:__ - __:__, 2 – no
	Wednesday	1 – yes, __:__ - __:__, 2 - no	1 – yes, __:__ - __:__, 2 – no
	Thursday	1 – yes, __:__ - __:__, 2 - no	1 – yes, __:__ - __:__, 2 – no
	Friday	1 – yes, __:__ - __:__, 2 - no	1 – yes, __:__ - __:__, 2 – no
	Saturday	1 – yes, __:__ - __:__, 2 - no	1 – yes, __:__ - __:__, 2 – no
	Sunday	1 – yes, __:__ - __:__, 2 - no	1 – yes, __:__ - __:__, 2 – no
17	Minimum number of SWs at the hotspot?	persons:	persons:
18	Maximum number of SWs at the hotspot?	persons:	persons:
19	Minimum age of SWs at the hotspot?	years:	years:
20	Maximum age of SWs at the hotspot?	years:	years:
21	Rate the safety of the hotspot, using a 5-point scale	1 – very unsafe<br>2 – fairly unsafe<br>3 – 50/50<br>4 – fairly safe<br>5 – very safe	1 – very unsafe<br>2 – fairly unsafe<br>3 – 50/50<br>4 – fairly safe<br>5 – very safe
22	Can the interviewer access the hotspot without restriction?	1 – yes<br>2 – no, who could help? ____<br>3 – don't know	Question is filed only at L1
23	Hotspot features (if applicable)		
25	What other hotspot types do SWs from this hotspot also work at?*		
26	What other city areas do SWs from this hotspot also work in?		
27	Is this hotspot included in HIV prevention program lists?	1 – yes, included<br>2 – no, new hotspot	1 – yes, included<br>2 – no, new hotspot
**QUESTIONS ABOUT THE SW HOTSPOT – LEVEL 2 ONLY**			
28	Are HIV prevention services available at this hotspot? (e.g., condom distribution, harm reduction counseling, rapid testing, etc.)		1 – yes, how many times in the past month? _____<br>2 –no<br>3 – don't know
29	Does a mobile van visit the hotspot to provide services? (e.g., condom distribution, harm reduction counseling, rapid testing, etc.)		1 – yes, how many times in the past month? _____

		2 –no<br>3 – don't know
30	Are there people who inject drugs at the hotspot?	1 – yes, persons: _________<br>2 – no
31	Are there male SWs at the hotspot?	1 – yes, persons: _________
32	Are there transgender SWs at the hotspot?	2 – no
33	Number of SWs present at the hotspot during validation	persons:
34	Do you know other hotspots in the city where SWs work?	1 – yes (if not in the general list, fill out a new MAPPING level form)<br>2 – no
35	We plan to conduct a study on sexual behavior and HIV prevalence among SWs. Would you agree to participate and take tests for HIV and hepatitis C?	1 – yes<br>2 – no<br>3 – don't know
36	Did a gatekeeper accompany you to the hotspot?	1 – yes, indicate type**<br>2 – no
37	Is information about your services available on other platforms/resources?	1 – yes<br>2 – no<br>3 – don't know
38	Specify the addresses of hotspot where information about your services is available	

39. Validation date and result:

Visit No	Date	Result (active or not)	Comment
First			
Second			
Third			

*HOTSPOT TYPE		**KEY INFORMANT TYPE	
1	Office ('brothel')	1	Representatives of NGOs working with the SW
2	Street, park, square, etc.	2	AIDS Center and social services staff
3	Route, highway	3	Tax/truck drivers
4	Nightclubs, casinos, discos, etc.	4	Law enforcement officers
5	Cafes, bars, restaurants, etc.	5	Pimps/hotspot managers
6	Massage parlor, sauna, bath	6	Entertainment hotspot staff (bartenders, waiters, security guards, etc.)
7	Art clubs/strip bars	7	Hotel personnel (bartenders, security guards, administrators, etc.)
8	Hotel/motel	8	Massage parlor/sauna staff
9	Apartment (own, rented)	9	Sex workers
10	Escort/on-call	10	Other KIs that can be recommended during the survey (indicate who exactly:
11	Virtual hotspot (internet)		
12	Printed ads in newspapers, magazines		
13	Pimps		
