# Supplementary material for: Mapping of sex work hotspots to guide targeted HIV prevention: Evidence from eight Ukrainian cities
Source: PLoS One. 2026 Feb 26;21(2):e0343803. doi: 10.1371/journal.pone.0343803 (PMC12944754; doi:10.1371/journal.pone.0343803)
Supplement: S1 Table — (PDF) [file pone.0343803.s002.pdf]

S1 Table. Characteristics of Sex Work Hotspots in Eight Ukrainian Cities, 2021

Characteristics	Dnipro	Kharkiv	Lviv	Kyiv	Odesa	Mariupol	Kropyvnytskyi	Cherkasy
	%	%	%	%	%	%	%	%
Reported hotspots, N	523	395	401	430	306	231	159	136
Validated active, %	75.1	97.0	79.3	72.6	92.8	85.7	73.6	83.1
**Total active, N (%)**	**393**	**383**	**318**	**312**	**284**	**198**	**117**	**113**
**Hotspot type**								
Apartment	32.8	67.4	28.6	53.8	54.9	38.4	5.1	27.4
Virtual hotspot	0.3	10.2	17.0	23.4	9.2	1.0	23.9	18.6
Street/park	9.2	11.0	3.1	8.3	21.1	18.2	9.4	13.3
Escort/on-call	0.3	0.8	35.8	-	-	-	13.7	-
Massage parlor/sauna	13.7	1.0	0.6	-	0.4	18.2	17.9	6.2
Café/ bar	16.0	-	0.3	-	2.1	9.6	8.5	15.9
Hotel/motel	9.9	2.6	3.5	1.0	3.5	10.6	8.5	8.8
Office ('brothel')	11.5	2.3	-	7.1	0.7	-	-	-
Nightclub/casino	2.8	1.0	0.9	1.0	2.5	3.0	1.7	4.4
Route/ highway	1.8	1.0	1.3	4.2	4.6	1.0	10.3	5.3
Pimps	-	0.5	8.2	-	-	-	0.9	-
Art club/strip club	1.8	1.0	0.6	0.3	1.1	-	-	-
**Subgroup presence**								
SWs who inject drugs	12.2	1.6	-	5.8	9.2	13.6	12.8	31.0
Male SWs	2.3	3.4	15.4	5.4	-	1.0	3.4	2.7
SEMs	10.7	0.5	0.6	1.6	-	29.8	0.9	13.3
**Age**								
Younger (max age ≤25 years)	13.5	2.1	36.8	8.3	10.6	3.0	10.3	15.0
Mixed (varied age ranges)	81.2	34.5	38.4	54.8	73.2	93.9	47.0	77.9
Older (min age >25 years)	5.3	63.4	24.8	36.9	16.2	3.0	42.7	7.1
**# SWs during L2-visit**								
≤3 SWs	53.9	62.9	72.0	60.9	28.2	55.6	52.1	54.9
4-9 SWs	43.5	33.7	23.6	34.0	64.4	44.4	42.7	40.7
≥10	2.5	3.4	4.4	5.1	7.4	-	5.1	4.4
**Season**								
Summer	99.7	100.0	99.7	100.0	99.6	100.0	100.0	97.3
Spring	100.0	100.0	99.7	99.4	97.2	98.0	100.0	100.0
Autumn	98.5	100.0	1.3	99.4	98.9	88.9	90.6	100.0
Winter	96.2	100.0	95.3	98.7	96.8	79.8	87.2	89.4
**Operate year-round**	**96.2**	**100.0**	**95.0**	**98.7**	**95.4**	**79.8**	**87.2**	**86.7**

Characteristics	Dnipro	Kharkiv	Lviv	Kyiv	Odesa	Mariupol	Kropyvnytskyi	Cherkasy
	%	%	%	%	%	%	%	%
**Working days**								
Monday	97.5	99.7	95.6	91.0	79.2	90.4	99.1	100.0
Tuesday	98.0	100.0	95.6	92.9	98.9	99.5	99.1	100.0
Wednesday	98.5	100.0	95.6	94.6	100.0	99.5	99.1	100.0
Thursday	99.0	100.0	95.6	95.5	100.0	99.5	99.1	100.0
Friday	99.7	100.0	96.9	98.7	100.0	100.0	100.0	100.0
Saturday	99.2	99.7	99.7	94.9	100.0	100.0	100.0	96.5
Sunday	99.2	99.7	96.2	91.7	100.0	100.0	100.0	96.5
**24/7 without days off**	**67.9**	**79.9**	**73.3**	**27.2**	**44.7**	**64.1**	**63.2**	**36.3**
**Schedule pattern**								
Predominantly nighttime	14.5	15.4	25.2	41.3	46.1	23.7	33.3	41.6
Predominantly daytime	14.5	4.7	0.6	21.5	2.8	9.1	2.6	19.5
Balanced	71.0	79.9	74.2	37.2	51.1	67.2	64.1	38.9
**Median work hours (IQR)**								
Weekday	24 (12-24)	24 (24-24)	24 (13-24)	12 (8-24)	19 (6-24)	24 (10-24)	24 (10-24)	24 (24-24)
Weekend	24 (12-24)	24 (24-24)	24 (13-24)	12 (8-24)	24 (6-24)	24 (11-24)	24 (10-24)	11 (7-24)
**Open access**	**99.7**	**99.7**	**50.9**	**92.3**	**99.6**	**93.9**	**100.0**	**52.2**
**Safety level**								
Very or fairly safe	46.8	93.7	87.7	95.5	76.8	67.7	64.1	55.8
50/50	38.4	0.3	8.8	3.2	19.7	25.3	33.3	26.5
Very or fairly unsafe	14.8	6.0	3.5	1.3	3.5	7.1	2.6	17.7
**Cross-type hotspots**	**47.3**	**58.5**	**89.9**	**24.0**	**4.6**	**65.2**	**75.2**	**2.7**
**Registered with HIV program[1]**	**16.3**	**20.1**	**0.3**	**7.7**	**39.4**	**2.5**	**-**	**6.2**
**Reported HIV prevention coverage[2]**	**16.5**	**23.2**	**9.1**	**73.4**	**89.4**	**19.2**	**4.3**	**12.4**
**Reported HIV mobile van coverage[2]**	**15.5**	**12.8**	**5.0**	**10.9**	**70.4**	**12.6**	**2.6**	**10.6**
**HIV prevention category**								
Reported & registered	16.3	17.0	-	7.4	39.4	2.0	-	4.4
Reported & not registered	0.3	6.3	9.1	66.0	50.0	17.2	4.3	8.0
Not reported & registered	-	3.1	0.3	0.3	-	0.5	-	1.8
Not reported & not registered	83.5	73.6	90.6	26.3	10.6	80.3	95.7	85.8

[1] 'Registered with HIV program' indicates hotspots officially registered as recipients of HIV-prevention services in 2020.

[2] 'Reported HIV prevention coverage' and 'Reported HIV mobile van coverage' refers to hotspots where key informants reported any form of HIV-prevention service during the month prior to the visit.

L2 – Level 2; SEM – sexually exploited minor; SW – sex worker
