# Supplementary material for: Mapping of sex work hotspots to guide targeted HIV prevention: Evidence from eight Ukrainian cities
Source: PLoS One. 2026 Feb 26;21(2):e0343803. doi: 10.1371/journal.pone.0343803 (PMC12944754; doi:10.1371/journal.pone.0343803)
Supplement: S2 Table — (PDF) [file pone.0343803.s003.pdf]

# S2 Table. Heatmap of Hourly Activity by Hotspot Type in Eight Ukrainian Cities, 2021

| Hotspot Type, N (Q1/Q2/Q3) | 00:00–01:00 | 01:00–02:00 | 02:00–03:00 | 03:00–04:00 | 04:00–05:00 | 05:00–06:00 | 06:00–07:00 | 07:00–08:00 | 08:00–09:00 | 09:00–10:00 | 10:00–11:00 | 11:00–12:00 | 12:00–13:00 | 13:00–14:00 | 14:00–15:00 | 15:00–16:00 | 16:00–17:00 | 17:00–18:00 | 18:00–19:00 | 19:00–20:00 | 20:00–21:00 | 21:00–22:00 | 22:00–23:00 | 23:00–24:00 |
|---|---|---|---|---|---|---|---|---|---|---|---|---|---|---|---|---|---|---|---|---|---|---|---|---|
| **Apartment, N=915 (74.3/81.4/87.5)** | | | | | | | | | | | | | | | | | | | | | | | | |
| Monday | 81.9 | 81.1 | 76.5 | 75.2 | 73.3 | 71.3 | 71.4 | 68.5 | 68.1 | 69,5 | 72.1 | 74.0 | 78.0 | 78.9 | 82.0 | 82.3 | 83.7 | 84.6 | 87.4 | 88.5 | 91.4 | 91.6 | 89.9 | 87.9 |
| Tuesday | 84.0 | 83.3 | 78.6 | 77.2 | 75.3 | 73.2 | 70.6 | 70.4 | 69.8 | 71.3 | 73.9 | 75.8 | 80.0 | 80.8 | 83.8 | 84.3 | 85.8 | 86.8 | 89.7 | 90.9 | 94.0 | 94.2 | 92.6 | 90.5 |
| Wednesday | 84.5 | 83.7 | 79.0 | 77.5 | 75.5 | 73.3 | 70.7 | 70.5 | 70.1 | 71.5 | 74.1 | 76.0 | 80.0 | 80.8 | 83.8 | 84.3 | 85.8 | 86.8 | 89.9 | 91.1 | 94.2 | 94.4 | 92.8 | 90.7 |
| Thursday | 84.7 | 83.9 | 79.2 | 77.7 | 75.7 | 73.6 | 70.9 | 70.7 | 70.2 | 71.6 | 74.2 | 76.2 | 80.3 | 81.1 | 84.2 | 84.6 | 86.1 | 87.1 | 90.2 | 91.5 | 94.6 | 94.9 | 93.2 | 91.1 |
| Friday | 85.2 | 84.5 | 79.7 | 78.1 | 76.2 | 74.0 | 71.5 | 71.3 | 70.7 | 72.1 | 74.8 | 76.7 | 81.0 | 81.7 | 84,8 | 85.1 | 86.7 | 87.8 | 90.9 | 92.1 | 95.0 | 95.2 | 93.6 | 91.5 |
| Saturday | 85.1 | 84.4 | 79,7 | 78.3 | 76.2 | 73.9 | 71.4 | 71.1 | 70.5 | 71.9 | 74.3 | 76.1 | 80.0 | 80.7 | 83.5 | 83.8 | 85.1 | 86.3 | 89.7 | 91.0 | 94.0 | 94.4 | 92.8 | 90.9 |
| Sunday | 85.0 | 84.3 | 79.7 | 78.3 | 76.1 | 73.8 | 71.3 | 71.0 | 70.4 | 71.1 | 74.1 | 75.7 | 79.5 | 80.1 | 82.7 | 83.1 | 84.0 | 85.2 | 88.6 | 89.9 | 93.0 | 93.6 | 92.0 | 90.3 |
| **Virtual hotspot, N=247 (84.2/86.6/90.3)** | | | | | | | | | | | | | | | | | | | | | | | | |
| Monday | 87.9 | 87.4 | 85.8 | 84.2 | 83.4 | 82.2 | 81.8 | 80.2 | 79.8 | 79.8 | 82.2 | 82.2 | 83.4 | 83.4 | 83.4 | 84.2 | 85.4 | 85.8 | 87.9 | 88.7 | 90.3 | 90.3 | 91.5 | 90.3 |
| Tuesday | 88.3 | 88.3 | 86.6 | 85.0 | 84.2 | 83.0 | 81.4 | 81.0 | 80.6 | 80.6 | 83.0 | 83.0 | 84.6 | 84.6 | 84.6 | 85.4 | 86.6 | 87.0 | 89.1 | 89.9 | 91.5 | 91.1 | 91.9 | 91.1 |
| Wednesday | 89.1 | 89.1 | 87.4 | 85.8 | 85.0 | 83.8 | 82.2 | 81.8 | 81.4 | 81.4 | 83.8 | 83.8 | 85.4 | 85.4 | 85.4 | 86.2 | 87.4 | 87.9 | 89.9 | 90.7 | 92.7 | 91.9 | 92.7 | 91.9 |
| Thursday | 89.9 | 89.9 | 88.3 | 86.6 | 85.4 | 84.2 | 82.6 | 82.2 | 81.8 | 81.8 | 84.2 | 84.2 | 85.8 | 85.8 | 85.8 | 86.6 | 87.9 | 88.3 | 90.3 | 91.1 | 93.1 | 92.3 | 93.5 | 92.7 |
| Friday | 91.1 | 91.1 | 89.1 | 87.9 | 86.6 | 85.4 | 82.6 | 82.2 | 82.2 | 82.2 | 84.6 | 84.6 | 86.2 | 86.2 | 86.2 | 87.0 | 87.9 | 88.3 | 90.3 | 91.5 | 93.5 | 92.7 | 93.5 | 92.7 |
| Saturday | 91.1 | 91.1 | 89.5 | 88.7 | 87.4 | 86.2 | 83.4 | 83.0 | 83.0 | 83.0 | 85.4 | 85.4 | 86.6 | 86.6 | 86.6 | 87.4 | 87.9 | 88.3 | 90.3 | 91.1 | 93.1 | 92.7 | 93.1 | 92.3 |
| Sunday | 91.1 | 91.1 | 90.3 | 89.5 | 88.3 | 87.0 | 84.2 | 83.8 | 83.4 | 83.8 | 85.8 | 85.8 | 86.6 | 86.6 | 86.6 | 87.4 | 87.9 | 88.3 | 89.9 | 91.1 | 92.3 | 92.3 | 93.1 | 92.3 |
| **Street/park, N=236 (14.3/30.1/52.2)** | | | | | | | | | | | | | | | | | | | | | | | | |
| Monday | 52.1 | 45.8 | 32.3 | 22.5 | 12.3 | 11.9 | 11.4 | 10.6 | 11.0 | 11.4 | 13.1 | 14.4 | 21.2 | 23.7 | 26.7 | 30.1 | 35.6 | 37.3 | 43.6 | 46.2 | 58.5 | 71.2 | 72.9 | 71.2 |
| Tuesday | 58.1 | 50.8 | 36.0 | 25.4 | 13.6 | 12.3 | 11.0 | 11.0 | 11.4 | 11.4 | 14.0 | 15.3 | 23.3 | 25.8 | 29.2 | 33.1 | 39.0 | 41.9 | 48.7 | 51.7 | 65.7 | 79.2 | 81.4 | 78.8 |
| Wednesday | 58.1 | 51.3 | 36.4 | 25.8 | 13.6 | 12.3 | 11.0 | 11.0 | 11.4 | 11.0 | 14.0 | 15.3 | 23.3 | 25.8 | 29.2 | 33.1 | 39.0 | 41.9 | 49.2 | 52.5 | 66.5 | 80.9 | 81.8 | 79.7 |
| Thursday | 58.5 | 52.1 | 37.3 | 26.7 | 14.0 | 12.7 | 11.4 | 11.4 | 11.9 | 11.4 | 14.4 | 15.7 | 23.7 | 26.3 | 29.7 | 33.9 | 39.8 | 42.8 | 49.6 | 53.0 | 66.9 | 81.4 | 82.2 | 80.1 |
| Friday | 59.7 | 53.4 | 38.1 | 27.5 | 14.8 | 13.6 | 11.9 | 11.9 | 12.3 | 11.9 | 14.4 | 15.7 | 23.7 | 26.3 | 30.1 | 33.9 | 39.8 | 43.2 | 50.8 | 53.8 | 68.2 | 82.2 | 83.5 | 80.5 |
| Saturday | 59.7 | 53.4 | 39.0 | 28.0 | 14.8 | 13.6 | 11.9 | 11.9 | 12.3 | 11.9 | 14.4 | 15.7 | 23.7 | 26.3 | 30.1 | 33.9 | 39.8 | 43.2 | 50.8 | 53.8 | 68.2 | 83.1 | 84.3 | 81.4 |
| Sunday | 59.7 | 53.0 | 38.6 | 27.5 | 14.8 | 13.6 | 11.9 | 11.9 | 12.3 | 12.3 | 14.4 | 15.7 | 23.7 | 26.3 | 30.1 | 33.9 | 39.8 | 43.2 | 50.4 | 53.8 | 68.2 | 83.1 | 84.3 | 81.4 |
| **Escort/ on-call, N=134 (94.8/96.3/98.0)** | | | | | | | | | | | | | | | | | | | | | | | | |
| Monday | 100.0 | 98.5 | 98.5 | 97.8 | 96.3 | 96.3 | 96.3 | 94.8 | 94.8 | 94.8 | 94.8 | 94.8 | 94.8 | 94.8 | 94.8 | 94.8 | 94.8 | 95.5 | 96.3 | 97.0 | 97.8 | 99.3 | 100.0 | 100.0 |
| Tuesday | 100.0 | 98.5 | 98.5 | 97.8 | 96.3 | 96.3 | 94.8 | 94.8 | 94.8 | 94.8 | 94.8 | 94.8 | 94.8 | 94.8 | 94.8 | 94.8 | 94.8 | 95.5 | 96.3 | 97.0 | 97.8 | 99.3 | 100.0 | 100.0 |
| Wednesday | 100.0 | 98.5 | 98.5 | 97.8 | 96.3 | 96.3 | 94.8 | 94.8 | 94.8 | 94.8 | 94.8 | 94.8 | 94.8 | 94.8 | 94.8 | 94.8 | 94.8 | 95.5 | 96.3 | 97.0 | 97.8 | 99.3 | 100.0 | 100.0 |
| Thursday | 100.0 | 98.5 | 98.5 | 97.8 | 96.3 | 96.3 | 94.8 | 94.8 | 94.8 | 94.8 | 94.8 | 94.8 | 94.8 | 94.8 | 94.8 | 94.8 | 94.8 | 95.5 | 96.3 | 97.0 | 97.8 | 99.3 | 100.0 | 100.0 |
| Friday | 100.0 | 98.5 | 98.5 | 97.8 | 96.3 | 96.3 | 94.8 | 94.8 | 94.8 | 94.8 | 94.8 | 94.8 | 94.8 | 94.8 | 94.8 | 94.8 | 94.8 | 95.5 | 96.3 | 97.0 | 97.8 | 99.3 | 100.0 | 100.0 |
| Saturday | 100.0 | 98.5 | 98.5 | 97.8 | 96.3 | 96.3 | 94.8 | 94.8 | 94.8 | 94.8 | 94.8 | 94.8 | 94.8 | 94.8 | 94.8 | 94.8 | 94.8 | 95.5 | 96.3 | 97.0 | 97.8 | 99.3 | 100.0 | 100.0 |
| Sunday | 100.0 | 98.5 | 98.5 | 97.8 | 96.3 | 96.3 | 94.8 | 94.8 | 94.8 | 94.8 | 94.8 | 94.8 | 94.8 | 94.8 | 94.8 | 94.8 | 94.8 | 95.5 | 96.3 | 97.0 | 97.8 | 99.3 | 100.0 | 100.0 |
| **Massage parlor/sauna, N=125 (85.4/89.6/92.8)** | | | | | | | | | | | | | | | | | | | | | | | | |
| Monday | 88.0 | 86.4 | 85.6 | 84.8 | 84.8 | 84.0 | 84.0 | 83.2 | 84.8 | 86.4 | 87.2 | 88.0 | 90.4 | 90.4 | 90.4 | 90.4 | 90.4 | 93.6 | 94.4 | 96.8 | 96.8 | 95.2 | 95.2 | 92.0 |
| Tuesday | 88.0 | 86.4 | 85.6 | 84.8 | 84.8 | 84.0 | 84.0 | 83.2 | 84.8 | 86.4 | 87.2 | 88.0 | 90.4 | 90.4 | 90.4 | 90.4 | 92.8 | 96.0 | 96.8 | 99.2 | 99.2 | 97.6 | 97.6 | 93.6 |
| Wednesday | 88.0 | 86.4 | 85.6 | 84.8 | 84.8 | 84.0 | 84.0 | 83.2 | 84.8 | 86.4 | 87.2 | 88.0 | 90.4 | 90.4 | 90.4 | 90.4 | 90.4 | 93.6 | 94.4 | 96.8 | 96.8 | 95.2 | 97.6 | 93.6 |
| Thursday | 88.0 | 86.4 | 85.6 | 84.8 | 84.8 | 84.0 | 84.0 | 83.2 | 84.8 | 86.4 | 87.2 | 88.0 | 90.4 | 90.4 | 90.4 | 90.4 | 92.8 | 96.0 | 96.8 | 99.2 | 99.2 | 97.6 | 97.6 | 93.6 |
| Friday | 88.0 | 86.4 | 85.6 | 84.8 | 84.8 | 84.0 | 84.0 | 83.2 | 84.8 | 86.4 | 87.2 | 88.0 | 90.4 | 90.4 | 90.4 | 90.4 | 90.4 | 93.6 | 94.4 | 96.8 | 96.8 | 95.2 | 98.4 | 94.4 |
| Saturday | 88.0 | 86.4 | 85.6 | 84.8 | 84.8 | 84.0 | 84.0 | 83.2 | 84.8 | 86.4 | 87.2 | 88.0 | 90.4 | 90.4 | 90.4 | 90.4 | 90.4 | 93.6 | 94.4 | 96.8 | 99.2 | 99.2 | 98.4 | 94.4 |
| Sunday | 88.0 | 86.4 | 85.6 | 84.8 | 84.8 | 84.0 | 84.0 | 83.2 | 84.8 | 86.4 | 87.2 | 88.0 | 90.4 | 90.4 | 90.4 | 90.4 | 90.4 | 93.6 | 94.4 | 96.8 | 99.2 | 98.4 | 97.6 | 93.6 |

| **Hotspot Type, N (Q1/Q2/Q3)** | **00:00–01:00** | **01:00–02:00** | **02:00–03:00** | **03:00–04:00** | **04:00–05:00** | **05:00–06:00** | **06:00–07:00** | **07:00–08:00** | **08:00–09:00** | **09:00–10:00** | **10:00–11:00** | **11:00–12:00** | **12:00–13:00** | **13:00–14:00** | **14:00–15:00** | **15:00–16:00** | **16:00–17:00** | **17:00–18:00** | **18:00–19:00** | **19:00–20:00** | **20:00–21:00** | **21:00–22:00** | **22:00–23:00** | **23:00–24:00** |
|---|---|---|---|---|---|---|---|---|---|---|---|---|---|---|---|---|---|---|---|---|---|---|---|---|
| **Café/bar, N=121 (15.3/45.9/68.2)** | | | | | | | | | | | | | | | | | | | | | | | | |
| Monday | 24.0 | 23.1 | 15.7 | 12.4 | 10.7 | 9.1 | 9.1 | 10.7 | 14.0 | 17.4 | 32.2 | 44.6 | 56.2 | 57.0 | 57.0 | 61.2 | 66.9 | 67.8 | 75.2 | 81.0 | 84.3 | 83.5 | 69.4 | 40.5 |
| Tuesday | 26.4 | 24.8 | 15.7 | 12.4 | 10.7 | 9.1 | 9.1 | 10.7 | 14.0 | 17.4 | 33.1 | 45.5 | 57.0 | 57.9 | 57.0 | 61.2 | 66.9 | 70.2 | 81.0 | 87.6 | 91.7 | 90.9 | 76.0 | 47.1 |
| Wednesday | 26.4 | 24.8 | 15.7 | 12.4 | 10.7 | 9.1 | 9.1 | 10.7 | 14.0 | 17.4 | 33.1 | 45.5 | 57.0 | 57.9 | 57.0 | 62.0 | 67.8 | 71.1 | 81.8 | 88.4 | 92.6 | 91.7 | 76.9 | 47.9 |
| Thursday | 26.4 | 24.8 | 15.7 | 12.4 | 10.7 | 9.1 | 9.1 | 10.7 | 14.0 | 17.4 | 33.1 | 45.5 | 57.0 | 57.9 | 57.0 | 62.0 | 67.8 | 71.1 | 81.8 | 88.4 | 92.6 | 91.7 | 76.9 | 47.9 |
| Friday | 26.4 | 24.8 | 16.5 | 13.2 | 10.7 | 9.1 | 9.1 | 10.7 | 14.0 | 17.4 | 33.9 | 46.3 | 58.7 | 59.5 | 58.7 | 63.6 | 69.4 | 72.7 | 83.5 | 90.1 | 94.2 | 93.4 | 78.5 | 47.9 |
| Saturday | 26.4 | 24.8 | 16.5 | 13.2 | 10.7 | 9.1 | 9.1 | 10.7 | 14.0 | 16.5 | 32.2 | 44.6 | 57.0 | 57.9 | 57.0 | 62.0 | 67.8 | 71.9 | 82.6 | 89.3 | 93.4 | 92.6 | 78.5 | 48.8 |
| Sunday | 26.4 | 24.8 | 16.5 | 13.2 | 10.7 | 9.1 | 9.1 | 10.7 | 14.0 | 16.5 | 32.2 | 44.6 | 57.0 | 57.9 | 57.0 | 62.0 | 67.8 | 71.9 | 82.6 | 89.3 | 93.4 | 92.6 | 78.5 | 47.9 |
| **Hotel/motel, N=114 (76.3/77.2/86.6)** | | | | | | | | | | | | | | | | | | | | | | | | |
| Monday | 91.2 | 91.2 | 84.2 | 81.6 | 81.6 | 78.1 | 78.1 | 74.6 | 74.6 | 74.6 | 74.6 | 75.4 | 77.2 | 77.2 | 77.2 | 77.2 | 78.1 | 78.1 | 82.5 | 85.1 | 93.9 | 96.5 | 98.2 | 95.6 |
| Tuesday | 91.2 | 91.2 | 84.2 | 81.6 | 81.6 | 77.2 | 74.6 | 73.7 | 74.6 | 74.6 | 74.6 | 75.4 | 76.3 | 76.3 | 76.3 | 76.3 | 77.2 | 77.2 | 82.5 | 85.1 | 94.7 | 97.4 | 99.1 | 97.4 |
| Wednesday | 91.2 | 91.2 | 84.2 | 81.6 | 81.6 | 77.2 | 74.6 | 73.7 | 74.6 | 74.6 | 74.6 | 75.4 | 77.2 | 76.3 | 76.3 | 76.3 | 77.2 | 77.2 | 82.5 | 84.2 | 93.9 | 96.5 | 98.2 | 96.5 |
| Thursday | 92.1 | 92.1 | 84.2 | 81.6 | 81.6 | 77.2 | 74.6 | 73.7 | 74.6 | 74.6 | 74.6 | 75.4 | 77.2 | 76.3 | 76.3 | 76.3 | 77.2 | 77.2 | 82.5 | 84.2 | 93.9 | 96.5 | 98.2 | 96.5 |
| Friday | 92.1 | 92.1 | 84.2 | 81.6 | 81.6 | 77.2 | 74.6 | 73.7 | 74.6 | 74.6 | 74.6 | 75.4 | 77.2 | 76.3 | 76.3 | 76.3 | 77.2 | 77.2 | 82.5 | 85.1 | 94.7 | 97.4 | 99.1 | 97.4 |
| Saturday | 92.1 | 92.1 | 84.2 | 81.6 | 81.6 | 77.2 | 74.6 | 73.7 | 74.6 | 74.6 | 74.6 | 75.4 | 77.2 | 76.3 | 76.3 | 76.3 | 77.2 | 77.2 | 82.5 | 85.1 | 94.7 | 97.4 | 99.1 | 97.4 |
| Sunday | 92.1 | 92.1 | 84.2 | 81.6 | 81.6 | 77.2 | 74.6 | 73.7 | 74.6 | 74.6 | 74.6 | 75.4 | 77.2 | 76.3 | 76.3 | 76.3 | 77.2 | 77.2 | 82.5 | 85.1 | 94.7 | 97.4 | 99.1 | 97.4 |
| **Office ('brothel'), N=78 (91.0/91.0/92.3)** | | | | | | | | | | | | | | | | | | | | | | | | |
| Monday | 92.3 | 92.3 | 91.0 | 91.0 | 89.7 | 88.5 | 88.5 | 87.2 | 87.2 | 88.5 | 91.0 | 91.0 | 91.0 | 91.0 | 92.3 | 92.3 | 92.3 | 91.0 | 92.3 | 92.3 | 93.6 | 94.9 | 93.6 | 93.6 |
| Tuesday | 92.3 | 92.3 | 91.0 | 91.0 | 89.7 | 88.5 | 88.5 | 87.2 | 87.2 | 88.5 | 91.0 | 91.0 | 91.0 | 91.0 | 92.3 | 92.3 | 92.3 | 91.0 | 92.3 | 92.3 | 93.6 | 94.9 | 93.6 | 93.6 |
| Wednesday | 92.3 | 92.3 | 91.0 | 91.0 | 89.7 | 88.5 | 88.5 | 87.2 | 87.2 | 88.5 | 91.0 | 91.0 | 91.0 | 91.0 | 92.3 | 92.3 | 92.3 | 91.0 | 92.3 | 92.3 | 93.6 | 94.9 | 93.6 | 93.6 |
| Thursday | 92.3 | 92.3 | 91.0 | 91.0 | 89.7 | 88.5 | 88.5 | 87.2 | 87.2 | 88.5 | 91.0 | 91.0 | 91.0 | 91.0 | 92.3 | 92.3 | 92.3 | 91.0 | 92.3 | 92.3 | 93.6 | 94.9 | 93.6 | 93.6 |
| Friday | 92.3 | 92.3 | 91.0 | 91.0 | 89.7 | 88.5 | 88.5 | 87.2 | 87.2 | 88.5 | 91.0 | 91.0 | 91.0 | 91.0 | 92.3 | 92.3 | 92.3 | 91.0 | 92.3 | 92.3 | 93.6 | 94.9 | 93.6 | 93.6 |
| Saturday | 93.6 | 93.6 | 92.3 | 92.3 | 91.0 | 89.7 | 88.5 | 88.5 | 88.5 | 89.7 | 91.0 | 91.0 | 91.0 | 91.0 | 92.3 | 92.3 | 92.3 | 91.0 | 92.3 | 92.3 | 93.6 | 94.9 | 94.9 | 94.9 |
| Sunday | 93.6 | 93.6 | 92.3 | 92.3 | 91.0 | 89.7 | 88.5 | 88.5 | 88.5 | 89.7 | 91.0 | 91.0 | 91.0 | 91.0 | 92.3 | 92.3 | 92.3 | 91.0 | 92.3 | 92.3 | 93.6 | 94.9 | 94.9 | 94.9 |
| **Route/highway, N=61 (26.2/42.6/53.7)** | | | | | | | | | | | | | | | | | | | | | | | | |
| Monday | 50.8 | 45.9 | 31.1 | 24.6 | 21.3 | 21.3 | 21.3 | 19.7 | 19.7 | 23.0 | 26.2 | 31.1 | 37.7 | 39.3 | 42.6 | 45.9 | 49.2 | 49.2 | 57.4 | 57.4 | 60.7 | 68.9 | 72.1 | 62.3 |
| Tuesday | 50.8 | 45.9 | 31.1 | 24.6 | 21.3 | 21.3 | 19.7 | 19.7 | 26.2 | 31.1 | 34.4 | 39,3 | 41.0 | 41.0 | 42.6 | 45.9 | 49.2 | 49.2 | 63.9 | 63.9 | 65.6 | 75.4 | 78.7 | 68.9 |
| Wednesday | 50.8 | 45.9 | 32.8 | 26.2 | 21.3 | 21.3 | 19.7 | 19.7 | 26.2 | 31.1 | 34.4 | 39.3 | 44.3 | 41.0 | 42.6 | 45.9 | 49.2 | 49.2 | 63.9 | 63.9 | 65.6 | 73.8 | 78.7 | 68.9 |
| Thursday | 50.8 | 45.9 | 32.8 | 26.2 | 21.3 | 21.3 | 19.7 | 19.7 | 26.2 | 31.1 | 34.4 | 39.3 | 44.3 | 41.0 | 42.6 | 45.9 | 49.2 | 49.2 | 63.9 | 63.9 | 65.6 | 73.8 | 78.7 | 68.9 |
| Friday | 50.8 | 45.9 | 32.8 | 26.2 | 21.3 | 21.3 | 19.7 | 19.7 | 26.2 | 31.1 | 34.4 | 37.7 | 44.3 | 41.0 | 42.6 | 45.9 | 49.2 | 49.2 | 63.9 | 63.9 | 65.6 | 73.8 | 78.7 | 72.1 |
| Saturday | 52.5 | 45.9 | 34.4 | 26.2 | 21.3 | 21.3 | 19.7 | 19.7 | 26.2 | 31.1 | 34.4 | 36.1 | 41.0 | 37.7 | 41.0 | 45.9 | 49.2 | 49.2 | 63.9 | 63.9 | 65.6 | 73.8 | 78.7 | 72.1 |
| Sunday | 52.5 | 45.9 | 34.4 | 26.2 | 21.3 | 21.3 | 19.7 | 19.7 | 26.2 | 31.1 | 34.4 | 36.1 | 41.0 | 37.7 | 41.0 | 45.9 | 49.2 | 49.2 | 63.9 | 63.9 | 65.6 | 73.8 | 78.7 | 72.1 |
| **Nightclub/casino, N=41 (29.3/39.0/63.4)** | | | | | | | | | | | | | | | | | | | | | | | | |
| Monday | 48.8 | 46.3 | 41.5 | 39.0 | 34.1 | 29.3 | 29.3 | 24.4 | 24.4 | 24.4 | 24.4 | 24.4 | 29.3 | 29.3 | 29.3 | 31.7 | 36.6 | 39.0 | 48.8 | 51.2 | 58.5 | 65.9 | 68.3 | 65.9 |
| Tuesday | 65.9 | 63.4 | 56.1 | 53.7 | 39.0 | 31.7 | 24.4 | 24.4 | 24.4 | 24.4 | 24.4 | 24.4 | 29.3 | 29.3 | 29.3 | 31.7 | 36.6 | 39.0 | 48.8 | 51.2 | 58.5 | 75.6 | 85.4 | 82.9 |
| Wednesday | 70.7 | 68.3 | 61.0 | 58.5 | 41.5 | 31.7 | 24.4 | 24.4 | 24.4 | 24.4 | 24.4 | 24.4 | 29.3 | 29.3 | 29.3 | 31.7 | 39.0 | 41.5 | 51.2 | 56.1 | 63.4 | 80.5 | 90.2 | 87.8 |
| Thursday | 70.7 | 68.3 | 61.0 | 58.5 | 41.5 | 31.7 | 24.4 | 24.4 | 24.4 | 24.4 | 24.4 | 24.4 | 29.3 | 29.3 | 29.3 | 31.7 | 39.0 | 41.5 | 51.2 | 56.1 | 63.4 | 80.5 | 90.2 | 87.8 |
| Friday | 78.0 | 75.6 | 68.3 | 65.9 | 48.8 | 36.6 | 24.4 | 24.4 | 24.4 | 24.4 | 24.4 | 24.4 | 29.3 | 29.3 | 29.3 | 31.7 | 39.0 | 43.9 | 53.7 | 58.5 | 65.9 | 82.9 | 95.1 | 95.1 |
| Saturday | 78.0 | 75.6 | 70.7 | 65.9 | 48.8 | 36.6 | 24.4 | 24.4 | 24.4 | 24.4 | 24.4 | 24.4 | 29.3 | 29.3 | 31.7 | 34.1 | 39.0 | 43.9 | 53.7 | 58.5 | 65.9 | 82.9 | 92.7 | 95.1 |
| Sunday | 80.5 | 78.0 | 73.2 | 65.9 | 48.8 | 36.6 | 24.4 | 24.4 | 24.4 | 24.4 | 24.4 | 24.4 | 29.3 | 29.3 | 31.7 | 34.1 | 39.0 | 43.9 | 53.7 | 58.5 | 65.9 | 82.9 | 95.1 | 97.6 |
| **Pimps, N=29 (48.3/51.7/55.2)** | | | | | | | | | | | | | | | | | | | | | | | | |
| Monday | 55.2 | 55.2 | 55.2 | 55.2 | 55.2 | 55.2 | 55.2 | 48.3 | 48.3 | 48.3 | 48.3 | 48.3 | 48.3 | 48.3 | 48.3 | 48.3 | 48.3 | 48.3 | 48.3 | 48.3 | 48.3 | 51.7 | 55.2 | 55.2 |
| Tuesday | 55.2 | 55.2 | 55.2 | 55.2 | 55.2 | 55.2 | 48.3 | 48.3 | 48.3 | 48.3 | 48.3 | 48.3 | 48.3 | 48.3 | 48.3 | 48.3 | 48.3 | 48.3 | 48.3 | 48.3 | 48.3 | 51.7 | 55.2 | 55.2 |
| Wednesday | 55.2 | 55.2 | 55.2 | 55.2 | 55.2 | 55.2 | 48.3 | 48.3 | 48.3 | 48.3 | 48.3 | 48.3 | 48.3 | 48.3 | 48.3 | 48.3 | 48.3 | 48.3 | 48.3 | 48.3 | 48.3 | 51.7 | 55.2 | 55.2 |
| Thursday | 55.2 | 55.2 | 55.2 | 55.2 | 55.2 | 55.2 | 48.3 | 48.3 | 48.3 | 48.3 | 48.3 | 48.3 | 48.3 | 48.3 | 48.3 | 48.3 | 48.3 | 48.3 | 48.3 | 48.3 | 48.3 | 51.7 | 55.2 | 55.2 |

| Hotspot Type, N (Q1/Q2/Q3) | 00:00–01:00 | 01:00–02:00 | 02:00–03:00 | 03:00–04:00 | 04:00–05:00 | 05:00–06:00 | 06:00–07:00 | 07:00–08:00 | 08:00–09:00 | 09:00–10:00 | 10:00–11:00 | 11:00–12:00 | 12:00–13:00 | 13:00–14:00 | 14:00–15:00 | 15:00–16:00 | 16:00–17:00 | 17:00–18:00 | 18:00–19:00 | 19:00–20:00 | 20:00–21:00 | 21:00–22:00 | 22:00–23:00 | 23:00–24:00 |
|---|---|---|---|---|---|---|---|---|---|---|---|---|---|---|---|---|---|---|---|---|---|---|---|---|
| Friday | 69.0 | 69.0 | 69.0 | 69.0 | 69.0 | 69.0 | 51.7 | 51.7 | 51.7 | 51.7 | 51.7 | 51.7 | 51.7 | 51.7 | 51.7 | 51.7 | 51.7 | 51.7 | 51.7 | 51.7 | 51.7 | 55.2 | 69.0 | 69.0 |
| Saturday | 100.0 | 100.0 | 100.0 | 100.0 | 100.0 | 100.0 | 55.2 | 55.2 | 55.2 | 55.2 | 55.2 | 55.2 | 55.2 | 55.2 | 55.2 | 55.2 | 55.2 | 55.2 | 55.2 | 55.2 | 55.2 | 58.6 | 100.0 | 100.0 |
| Sunday | 58.6 | 58.6 | 58.6 | 58.6 | 58.6 | 58.6 | 51.7 | 51.7 | 51.7 | 51.7 | 51.7 | 51.7 | 51.7 | 51.7 | 51.7 | 51.7 | 51.7 | 51.7 | 51.7 | 51.7 | 51.7 | 55.2 | 58.6 | 58.6 |
| **Art club/strip club, N=17 (29.4/41.2/70.6)** | | | | | | | | | | | | | | | | | | | | | | | | |
| Monday | 76,5 | 76,5 | 76,5 | 70,6 | 70,6 | 41.2 | 41.2 | 29.4 | 29.4 | 29.4 | 29.4 | 29.4 | 29.4 | 29.4 | 29.4 | 35.3 | 35.3 | 35.3 | 35.3 | 35.3 | 52.9 | 58,8 | 76.5 | 76.5 |
| Tuesday | 94.1 | 88.2 | 76.5 | 70.6 | 70.6 | 41.2 | 29.4 | 29.4 | 29.4 | 29.4 | 29.4 | 29.4 | 29.4 | 29.4 | 29.4 | 35.3 | 35.3 | 35.3 | 35.3 | 35.3 | 52.9 | 64.7 | 88.2 | 94.1 |
| Wednesday | 94.1 | 88.2 | 76.5 | 70.6 | 70.6 | 41.2 | 29.4 | 29.4 | 29.4 | 29.4 | 29.4 | 29.4 | 29.4 | 29.4 | 29.4 | 35.3 | 35.3 | 41.2 | 41.2 | 41.2 | 58.8 | 70.6 | 94.1 | 100.0 |
| Thursday | 94.1 | 88.2 | 76.5 | 70.6 | 70.6 | 41.2 | 29.4 | 29.4 | 29.4 | 29.4 | 29.4 | 29.4 | 29.4 | 29.4 | 29.4 | 35.3 | 35.3 | 41.2 | 41.2 | 41.2 | 58.8 | 70.6 | 94.1 | 100.0 |
| Friday | 94.1 | 88.2 | 76.5 | 70.6 | 70.6 | 41.2 | 29.4 | 29.4 | 29.4 | 29.4 | 29.4 | 29.4 | 29.4 | 29.4 | 29.4 | 35.3 | 35.3 | 41.2 | 41.2 | 41.2 | 58.8 | 70.6 | 94.1 | 100.0 |
| Saturday | 94.1 | 88.2 | 76.5 | 70.6 | 70.6 | 41.2 | 29.4 | 29.4 | 29.4 | 29.4 | 29.4 | 29.4 | 29.4 | 29.4 | 29.4 | 35.3 | 35.3 | 41.2 | 41.2 | 41.2 | 58.8 | 70.6 | 94.1 | 100.0 |
| Sunday | 94.1 | 88.2 | 76.5 | 70.6 | 70.6 | 41.2 | 29.4 | 29.4 | 29.4 | 29.4 | 29.4 | 29.4 | 29.4 | 29.4 | 29.4 | 35.3 | 35.3 | 41.2 | 41.2 | 41.2 | 58.8 | 70.6 | 94.1 | 100.0 |

Q1, Q2, and Q3 are expressed as percentages and represent the 25th, 50th (median), and 75th percentiles of the 'Percent_Open' values over all 168 one-hour intervals. The four cell colors correspond to quartile ranges for each venue type: red (Very Low): Percent_Open≤Q1, orange (Low): Q1<Percent_Open≤Q2, light green (Moderate): Q2<Percent_Open<Q3, dark green (High): Percent_Open≥Q3. The color indicates whether the proportion of open hotspots for that type falls within the bottom 25% (red), the next 25% (orange for 25-50% and light green for 50-75%), or the top 25 % (dark green) of all hours for that category.
