# Supplementary material for: Mapping of sex work hotspots to guide targeted HIV prevention: Evidence from eight Ukrainian cities
Source: PLoS One. 2026 Feb 26;21(2):e0343803. doi: 10.1371/journal.pone.0343803 (PMC12944754; doi:10.1371/journal.pone.0343803)
Supplement: Attachment — Submitted filename: Response to Reviewers.pdf [file pone.0343803.s005.pdf]

Review comments for manuscript entitled "Mapping of sex work hotspots to guide targeted HIV prevention: evidence from eight Ukrainian cities"

Version: September 21, 2025

We thank the Editor and the reviewers for their thoughtful and constructive comments, which have improved the quality of the manuscript. All comments have been addressed in detail below. Reviewer comments are presented verbatim, followed by our responses in italicized text. A tracked-changes version and a clean revised manuscript are provided for ease of review.

Journal requirements:

1. Please ensure that your manuscript meets PLOS ONE's style requirements, including those for file naming. The PLOS ONE style templates can be found at

https://journals.plos.org/plosone/s/file?id=wjVg/PLOSOne_formatting_sample_main_body.pdf and

https://journals.plos.org/plosone/s/file?id=ba62/PLOSOne_formatting_sample_title_authors_affiliations.pdf

— *We thank the editors for this reminder. We have reviewed the PLOS ONE style and file naming requirements and updated all manuscript files to ensure consistency with the journal's formatting templates.*

2. Thank you for stating the following in the Sources of support Section of your manuscript: "This study was conducted with the financial support of the International Charitable Foundation "Alliance for Public Health" through the project "Improving HIV Treatment Cascade for Key Populations through Differentiated Case Detection and Linkage to Care and Increased Capacity at the Center for Public Health and Strategic Information in Ukraine", funded by the U.S. Centers for Disease Control and Prevention (CDC) through the US President's Emergency Plan for AIDS Relief (PEPFAR). This study is supported by the Cooperation Agreement NU2GGH002114 with the CDC. All responsibility for the content shall be borne by the authors, and the article does not necessarily reflect the official position of the CDC."

We note that you have provided funding information that is currently declared in your Funding Statement. However, funding information should not appear in the Acknowledgments section or other areas of your manuscript. We will only publish funding information present in the Funding Statement section of the online submission form.

Please remove any funding-related text from the manuscript and let us know how you would like to update your Funding Statement. Currently, your Funding Statement reads as follows: "This study was conducted with the financial support of the International Charitable Foundation "Alliance for Public Health" through the project "Improving HIV Treatment Cascade for Key Populations through Differentiated Case Detection and Linkage to Care and Increased Capacity at the Center for Public Health and Strategic Information in Ukraine", funded by the U.S. Centers for Disease Control and Prevention (CDC) through the US President's Emergency Plan for AIDS Relief (PEPFAR). This study is supported by the Cooperation Agreement NU2GGH002114 with the CDC. All responsibility for the content shall be borne by the authors, and the article does not necessarily reflect the official position of the CDC."

Please include your amended statements within your cover letter; we will change the online submission form on your behalf.

- *Thank you for this clarification. We confirm that all funding-related text has been removed from the manuscript body, and funding information is now provided exclusively in the Funding Statement in the online submission system, as requested.*

3. For studies involving third-party data, we encourage authors to share any data specific to their analyses that they can legally distribute. PLOS recognizes, however, that authors may be using third-party data they do not have the rights to share. When third-party data cannot be publicly shared, authors must provide all information necessary for interested researchers to apply to gain access to the data. (https://journals.plos.org/plosone/s/data-availability#loc-acceptable-data-access-restrictions)

For any third-party data that the authors cannot legally distribute, they should include the following information in their Data Availability Statement upon submission:

1) A description of the data set and the third-party source

2) If applicable, verification of permission to use the data set

3) Confirmation of whether the authors received any special privileges in accessing the data that other researchers would not have

4) All necessary contact information others would need to apply to gain access to the data

- *Thank you for this comment and for highlighting the journal's data availability requirements. We would like to clarify that this study did not use third-party data. All data were collected by the International Charitable Foundation "Alliance for Public Health" (APH), which is the data owner and holds the legal rights to use and share the datasets. However, the datasets generated and analyzed in this study contain sensitive information related to key populations and specific sex work locations. In accordance with APH data protection policies and ethical requirements, even without the personal identifiers the data cannot be made publicly available in order to protect participant confidentiality and safety. We have revised and clarified the Data Availability Statement to explicitly specify the data ownership, the reason for restricted access, and the procedure for requesting access to the data, including contact information (office@aph.org.ua), in line with PLOS ONE guidelines.*

Reviewer 1

Generally, the topic addresses a critical gap in Ukraine's HIV response, where the epidemic is concentrated among key populations like SWs. The study incorporates modern challenges like digitalization (e.g., virtual platforms at 11.7%), which aligns with post-2020 shifts due to COVID-19 and conflict. The two-stage multi-informant design (1,212 secondary + 2,277 primary informants) is robust and community-led, achieving high validation (86.8%). Analysis includes advanced stats (e.g., multivariable mixed-effects regression), which is appropriate for clustered data like hotspots. The method is sound. The study will generally have a Policy Impact if the findings are utilized. Emphasizes equity for vulnerable subgroups, which will provide a better UNAIDS 95-95-95 and leave no one behind.

However, there is a Lack of Novelty Clarity: While the study highlights "the most comprehensive mapping," it doesn't explicitly compare to prior work (e.g TAMPEP 2018 mapped FSW coverage at 40.3%). Without citing baselines, the contribution feels understated.

- *Thank you for this comment and for emphasizing the importance of clearly situating the study's contribution in relation to prior work. We would like to clarify that the figures cited from the TAMPEP report (e.g., 40.3% coverage) refer to* ***individual-level*** *programmatic coverage of female SWs in 2011, derived from aggregated service statistics recorded in the SYREX database. These data describe the proportion of*

individual FSWs reached by HIV prevention services at the population level at that time, rather than the coverage of SW hotspots or venues. Although the report is titled National Mapping on Sex Work, its analytical focus is on describing the structure and characteristics of FSWs in the country, rather than on systemic mapping, validation, or analysis of sex work locations.

In Ukraine, IBBS among SWs has been implemented regularly sine 2005, and hotspot mapping has traditionally been conducted as a preparatory step for IBBS sampling and PSE. However, such mapping exercises were typically characterized by low validation rates, limited documentation of hotspot characteristics, and minimal analytical use beyond sampling frame construction. Importantly, they were not designed to assess ***hotspot-level*** *HIV prevention service coverage or to examine how venue typologies and operational features shape service delivery. To our knowledge, no prior studies in Ukraine have systematically assessed the proportion of sex work hotspots covered by HIV prevention services, nor analyzed coverage in relation to hotspot characteristics such as venue type, access modality, operating schedule, perceived safety, or organizational structure. As a result, there is no historical baseline for* ***hotspot-level*** *coverage against which service reach across different sex work settings could be evaluated.*

Accordingly, the novelty of this study lies not in updating individual-level prevention coverage estimates, but in introducing a validated, multi-city, hotspot-level analytical framework that explicitly links sex work organization, venue typologies, and structural characteristics to HIV prevention service delivery. This approach allows identification of systematic and predictable service gaps – particularly in private, digital, and manager-controlled settings – that cannot be captured through individual-level coverage indicators alone. We have revised the Introduction to more explicitly articulate this distinction and to clarify that the absence of prior hotspot-level baselines reflects a genuine evidence gap, which this study is the first to address in the Ukrainian context (paragraphs 4-5 of the Introduction section).

The author needs to specify cities e.g Kyiv, Odesa?, sampling details (e.g., how informants were recruited), or ethical considerations e.g SW safety in conflict zones). Statistical methods are mentioned but not justified e.g why exploratory factor analysis?

- *Thank you for this comment. We believe that much of the requested information is already described in detail in the Methods section, but we appreciate the opportunity to clarify and provide additional details. First, we would like to clarify that the study cities are already specified in the Population and Settings section (Kyiv, Odesa, Cherkasy, Dnipro, Kharkiv, Kropyvnytskyi, Lviv, and Mariupol), together with the rationale for their selection based on historical HIV prevalence and population size estimates among sex workers.*

  With respect to safety and ethical considerations, it is important to clarify the temporal and contextual scope of the study. All data were collected in 2021, prior to the full-scale Russian invasion of Ukraine in February 2022. The study did not take place during active hostilities and did not conduct fieldwork in conflict-affected or frontline zones. While an armed conflict existed in eastern Ukraine before 2022, none of the study cities were located in active conflict zones at the time of data collection. Accordingly, ethical safeguards were designed to address risks related to criminalization, stigma, and policing rather than active warfare. These procedures – including IRB approvals, informed consent, anonymization, engagement of peer workers, and coordination with local authorities – are described in the Ethical Considerations section.

  At the same time, we have expanded the description of the sampling and recruitment of secondary KIs in the Level 1: Hotspot Identification subsection. We now describe the purposive selection of KIs based on their routine contact with sex work settings, the involvement of local NGOs and SW community representatives in developing initial informant lists, and the iterative expansion of informants through referrals until

saturation was reached (paragraphs 1-2 of the Level 1: hotspot identification subsection). Regarding statistical methods, we have strengthened the justification for the use of Exploratory Factor Analysis (EFA) in the Data Analysis section. We now explicitly explain that EFA was applied to address a specific analytical gap – namely, that single-category hotspot classifications do not adequately capture overlapping operational contexts of sex work relevant for outreach planning. The rationale for EFA and its linkage to the first research question are now articulated in more detail, alongside the justification for the multivariable GLMM used to address the second research question (paragraphs 3-4 of the Data analysis subsection).

On the results, Percentages are precise, but no confidence intervals or p-values, reducing interpretability.

— *We thank the reviewer for this suggestion, which we believe refers to Tables 2 and 3. We carefully considered the inclusion of confidence intervals (CIs) and p-values; however, we have decided to omit them for the following reasons.*

First, this study is not based on a sample but rather represents a theoretically complete census of the venues that were accessible in participating cities (N=2118). Our understanding of statistics is that CIs are used to estimate the degree of sampling error when generalizing from a sample to a population. Because our verified dataset contains the entire 'population' of venues, the sampling error is effectively zero, and the observed values (i.e., proportions) are the true population parameters.

Next, the primary objective of Table 2 is to present the numbers of key informants and venues identified by them, i.e. the table is intended to present the study process and not any population characteristics. Table 3 does present the characteristics of the venues 'population', however it is purely descriptive. We aim to provide an exact account of the population's characteristics during the study period rather than making inferences about a hypothetical "super-population" or future trends. Including CIs in a census can be mathematically redundant and potentially misleading to the reader, as it implies a level of uncertainty that does not exist within the scope of the census. Also, we did not intend to perform any statistical comparisons between the venue types (which would be impractical for twelve venue types) or make any inferences based on their characteristics, therefore Table 3 does not include any information about statistical tests or p-values.

We have added a sentence to the Materials and Methods section (paragraph 1 of the Data analysis subsection) to justify this approach: "As this study represents a full census of the target population, descriptive statistics are reported without confidence intervals or p-values, as the observed values represent the actual population parameters rather than sample estimates"

In Table 5 we do intend to make inferences about the association between the venue characteristics and prevention coverage, therefore we present regression output with adjusted odds ratios, 95% confidence intervals, and p-values.

On the results, ... Low coverage (e.g 13.7% registered) is highlighted, but no breakdown by city or subgroup size estimates.

We would also like to clarify that disaggregation of HIV prevention service coverage is already provided in the manuscript at two complementary levels. First, coverage is presented by hotspot type in Table 3, demonstrating variation across different operational settings (e.g., apartments, virtual hotspots, street-based locations). This typology-based presentation reflects how HIV prevention services are typically planned and delivered in practice and therefore provides programmatically relevant insight. Second, city-

level results are reported in the Supporting Information (S1 Table), where hotspot characteristics and HIV prevention coverage are shown separately for each of the eight study cities.

If the reviewer's comment refers to SW subgroups (rather than hotspot subgroups), then subgroup presence (e.g., SWs who inject drugs, male SWs, sexually exploited minors) was measured at the hotspot level and reflects reported co-occurrence of these populations within venues, rather than mutually exclusive subgroup sizes. Because multiple subgroups may be present at the same hotspot, and because service coverage was assessed at the hotspot rather than individual level, simple descriptive disaggregation by subgroup would be methodologically inappropriate and potentially misleading.

Presence of specific SW subgroups were measured as venue-level characteristics (reported in Table 3), and these variables were tested for potential association with HIV prevention coverage using multivariable GLMM, while accounting for other hotspot characteristics and city-level clustering. We have clarified this analytical rationale in the Methods and Discussion sections.

The paper Asserts "community-led mapping achieved high validation", but doesn't quantify community involvement. Recommendations e.g strong engagement with SW communities, are a bit generic without linking to specific findings.

- *Thank you for this important comment. We agree that statements regarding community-led mapping should be clearly supported by objective data. We would like to clarify that community involvement is quantified and documented in multiple sections of the manuscripts. Specifically, SWs constituted 45.6% of secondary KIs at Level 1 and 80.6% of primary KIs at Level 2. In addition, SWs were formally represented in all regional technical working groups and actively participated in planning and data collection. Hotspots reported by SWs demonstrated the highest validation rate (86.8%) compared to all other KI groups (Table 2), indicating greater accuracy in identifying active sex work locations. This finding directly underpins our assertion that community-led mapping achieved high validation and supports the recommendation for strong engagement with SW communities. At the same time, to address this suggestion, we have strengthened the Discussion to more explicitly link these quantitative findings to the programmatic recommendation, clarifying how community involvement contributed to improved data quality and identification of less visible hotspots (paragraph 7 of the Discussion section).*

There are Minor issues like inconsistent abbreviations (SWs vs. SW subgroups) and passive voice in places (e.g. "Data were analyzed" could be active for impact).

- *Thank you for this careful comment. We have ensured consistent use of abbreviations across the manuscript and reviewed the text for minor stylistic and typographical issues. Where appropriate, passive constructions were revised to active voice, including the example highlighted, to improve clarity and readability.*

Finally, no keywords or funding mention, common in abstracts.

- *Thank you for this comment. We would like to clarify that keywords and funding information were provided during manuscript submission in the designated fields of the journal's submission system and are also included on the second title page. The abstract was formatted strictly in accordance with the journal's author guidelines, which do not provide for the inclusion of keywords or funding statements within the abstract text. Moreover, following editorial guidance received during the review process, all funding information was excluded from the main manuscript body, including the abstract. We therefore did not add keywords or funding statements to the abstract in order to remain compliant with the journal's formatting and editorial requirements. However, we would be grateful if the Editorial Office could advise whether these elements should be added to the abstract to fully address this comment.*

Recommendations for Improvement

To strengthen this for submission, focus on clarity, depth, and enhance background: Add a sentence on Ukraine-specific context (e.g."Amid ongoing conflict, SW mobility has increased, complicating outreach"). Include a global benchmark (e.g."Global FSW HIV prevention coverage is approximately 50%, per UNAIDS").

- *Thank you for this suggestion. We have strengthened the Introduction by adding a brief Ukraine-specific contextual sentence highlighting the impact of ongoing conflict, displacement, and increased mobility of sex workers on outreach feasibility (paragraph 2 of the Introduction section). We also included a concise global framing statement to situate the study within broader evidence on uneven HIV prevention coverage among sex workers, particularly in less visible and digitally mediated settings (paragraph 4 of the Introduction section). These additions were intentionally kept concise to enhance contextual clarity without duplicating results or discussion content.*

Refine Methods: Specify cities, informant recruitment (e.g., snowball sampling), and ethics (e.g., IRB approval). Justify analyses (e.g., "Mixed-effects regression accounted for city-level clustering").

- *Thank you for this comment. We note that this point overlaps with the methodological comments provided earlier, and we have addressed those suggestions accordingly. Specifically, the study cities are specified in the Population and Settings section, and the description of informant recruitment has been expanded in the Level 1: Hotspot Identification subsection to clarify the purposive and iterative (referral-based) selection of secondary KIs until saturation. We have also strengthened the justification of analytical approaches in the Data Analysis section, explicitly explaining the rationale for both EFA and GLMM, including accounting for clustering at the city level. Regarding ethics, ethical considerations and IRB approval were already described in detail in the Ethical Considerations section of the manuscript. This includes approval by the Institutional Review Board of the Alliance for Public Health and review by the CDC, as well as procedures to ensure participant safety, confidentiality, and informed consent in a criminalized context. To avoid any ambiguity, we confirm that all data were collected in 2021, prior to the 2022 full-scale invasion, and the study did not involve active conflict zones.*

Add key stats with precision (e.g. say "Coverage OR=2.5 [95% CI:1.8-3.4] for safe hotspots").

- *Thank you for this suggestion. To improve interpretability without duplicating the full regression table in the text, we have added a small number of illustrative effects estimates directly to the Results section. Specifically, we now report adjusted odds ratios with 95% confidence intervals for selected key predictors (e.g., perceived safety and presence of SWs who inject drugs) (paragraph 6 of the Results section), while retaining Table 5 as the primary source for the complete set of regression results.*

Strengthen Conclusions: Link directly to results (e.g., "Gaps in virtual hotspots (11.7% of total) underscore digital outreach needs"). Add implications (e.g., "Scaling community-led mapping could increase coverage by 20-30%, per similar studies").

- *Thank you for this thoughtful suggestion. We agree that Conclusions should clearly reflect the key findings of the study. In revising this section, we aimed to strengthen the linkage to the results by explicitly highlighting the main structural gaps identified – particularly in less visible and digitally mediated sex work settings – and their implications for HIV prevention programming. At the same time, we intentionally avoided repeating specific numerical estimates or introducing new quantitative projections in the Conclusions, as detailed percentages and effect estimates are already presented in the Results and interpreted in the Discussion. We consider the Conclusions to serve primarily a synthetic and forward-looking function, focusing on the broader programmatic and policy implications of the findings rather that restating numerical results. We believe this approach preserves clarity, avoids redundancy, and aligns with conventional reporting standards for conclusion sections in studies.*

Overall, this is a solid paper with high public health relevance. With these tweaks, it could be publishable and impactful for HIV programs in conflict-affected areas.

Reviewer 2

General Comments

This manuscript, "Typologies of Sex Work Hotspots and Associations with HIV Prevention Coverage in Eight Ukrainian Cities: A Cross-Sectional Study," makes a valuable contribution to public health by characterizing sex work hotspots and their relationship to HIV service coverage in Ukraine. The methodology is sound, and the data provides significant value to researchers, policymakers, and public health practitioners. The manuscript is well-written, and the following suggestions are offered to strengthen it further.

Major Revisions

Alignment of Objectives, Methods, and Findings: The manuscript would benefit from a clearer alignment between objectives, methods, and findings. The introduction of Exploratory Factor Analysis (EFA) in the results feels abrupt. To improve coherence:

a. Explicitly state research questions at the end of the Introduction, e.g., "1) What are the operational typologies of sex work hotspots in urban Ukraine? 2) What hotspot characteristics are associated with HIV prevention service coverage?"

b. In the Data Analysis section, justify each statistical method by linking it to a specific research question. For example, state that EFA was used to answer question 1 by identifying latent structures among hotspot types, and the Generalized Linear Mixed Model (GLMM) was used to answer question 2.

- *Thank you for this suggestion. We revised the manuscript to strengthen alignment between the study objectives, analytical methods, and reported findings. Specifically, we added two explicit research questions at the end of the Introduction to clarify the analytical focus of the study (paragraph 5 of the Introduction section). In addition, the Data Analysis section was revised to link each statistical method to the corresponding research question: EFA was used to identify latent patterns in cross-type organization of sex work across hotspot types, while GLMM was applied to assess associations between hotspot characteristics and HIV prevention service coverage (paragraphs 3-4 of the Data analysis subsection). Importantly, EFA-defined venue types were used as the key independent variable in GLMM.*

Rationale and Integration of the Factor Analysis: Strengthen the rationale for EFA and integrate its results more fully into the discussion.

a. In the Methods, justify the importance of uncovering these underlying factors and the research gap this analysis fills.

- *Thank you for this valuable suggestion. We strengthened the methodological rationale for applying EFA by addressing the research gap it was intended to fill. In the Methods section, we now explain that single-category hotspot classifications may not adequately reflect overlapping operational contexts of sex work relevant that are critical for outreach planning. EFA was applied to cross-type hotspot data to identify latent operational settings that better capture shared access environments for HIV prevention delivery (paragraph 3 of the Data analysis subsection).*

b. Enhance the Discussion section by exploring the programmatic implications of the factors beyond restating them. For example, discuss what the "Hotel & Leisure Settings" factor implies for intervention design, such as a unified outreach strategy targeting venue owners or highlighting a specific risk environment.

– *We also revised the Discussion to move beyond simply restating factor structures and instead used the identified factors as an interpretive framework for programmatic implications. For example, the "Hotel & Leisure Settings" factor is discussed in relation to coordinated, venue-level engagement approaches across semi-formal indoor environments characterized by mediated access and management control (paragraph 2 of the Discussion section). Similar factor-based interpretation is applies to other settings (e.g., Club-Mediated Work) to explain patterns of service under-coverage, gatekeeping, and underreporting, thereby integrating the factor analysis throughout the Discussion rather than treating it as a standalone result.*

Strengthening the Discussion and Policy Implications: Strengthen the Discussion by drawing clearer connections between results and real-world implications.

a. Directly link specific findings from the regression analysis to actionable recommendations. For instance, for "Virtual + Escort/on-call" hotspots with extremely low coverage, recommend specific, evidence-based digital outreach strategies.

– *Thank you for this comment. We revised the Discussion to more clearly connect regression findings with their real-world implications, without repeating model outputs. Instead, we embedded these insights throughout the Discussion by explaining how hotspot characteristics associated with lower or higher service coverage translate to concrete programmatic responses. In particular, we strengthened interpretations related to Online & Independent Work, Hotel & Leisure Settings, and Club-Mediated Work environments, clarifying how structural access constraints, perceived safety, operating hours, and the presence of SWs who inject drugs inform outreach strategies under real-world conditions.*

b. Increase international relevance by comparing identified hotspot typologies with those found in other settings (e.g., other parts of Eastern Europe or globally).

– *We revised the Discussion to strengthen international contextualization across section. The identified hotspot typologies and access environments in Ukraine are now compared with evidence from other settings, including India and Mauritius (greater venue mobility), sub-Saharan Africa (limited reach of venue-based models among home-based and digitally mediated sex work), Central and Eastern Europe and Central Asia (COVID-19-related venue closures and shifts in work organization), and high-income contexts such as the UK. Rather than emphasizing country-specific differences alone, we frame these comparisons around shared structural mechanisms – such as visibility, gatekeeping, safety, criminalization, and digital mediation – that shape HIV service access across diverse settings.*

c. Sharpen policy messages with concrete recommendations for different stakeholders (e.g., NGOs, municipal health departments, law enforcement).

– *In revising the Discussion, we focused on strengthening policy relevance along three empirically grounded dimensions, rather than adding fragmented recommendations throughout the text. First, based on the consistently low coverage observed in Online & Independent Work settings, we highlight the need for national programs and NGOs to recognize digital outreach as a core HIV prevention modality, with corresponding implications for funding, guidance, and performance monitoring. Second, drawing on findings related to operating hours, perceived safety, and access modality, we emphasize that effective outreach must adapt to real working conditions of sex work rather than rely on fixed schedules or venue-based access assumptions, underscoring the value of flexible, community-informed and network-based outreach strategies. Third, we link these operational realities to gaps in routine monitoring systems, showing how off-site service delivery remains systematically underreported, with recommendations for national monitoring frameworks, including improved geolocation practices and accountability mechanisms. In addition to strengthening policy implications in the main Discussion, we also integrated stakeholder-relevant implications into the Limitations section, using method-related constraints (e.g.,*

invisibility of digital sex work, criminalization, temporal instability) as an entry point for outlining pragmatic adaptations in outreach, monitoring, and governance.

Minor Revisions

Clarity of the Regression Model in the Methods: In the Data Analysis section, clarify the hypothesis tested by the logistic regression model. Add a sentence stating that the model was used to identify which hotspot characteristics (independent variables) predict the likelihood of HIV prevention service coverage (the dependent variable).

- *Thank you for this suggestion. We clarified the purpose of the regression analysis in the Data Analysis section by explicitly stating that the model was used to identify which hotpot characteristics (independent variables) predict the likelihood of HIV prevention service coverage (dependent variable) (paragraph 4 of the Data analysis subsection).*

Presentation of Reference Groups in Tables: In Table 5, include the reference categories for predictors (e.g., "street/park" for Hotspot type) as a line item in the table with an assigned Odds Ratio of "1.00 (Reference)".

- *We revised Table 5 to present all reference categories as separate line items with an odds ratio of 1.00 (Reference), improving clarity and interpretability.*

Self-Contained Tables: Ensure all abbreviations (e.g., aOR, CI, KMO) and technical terms within the tables are defined in the footnotes of each respective table.

- *Thank you for this comment. All tables have been revised to be fully self-contained, with all abbreviations and technical terms defined in the corresponding table footnotes.*
